# The effect of recombinant versus plasma-derived von Willebrand factor on prolonged PFA closure times in ECMO patients with acquired von Willebrand syndrome – an observational study

**DOI:** 10.1186/s12959-022-00448-1

**Published:** 2023-01-10

**Authors:** Martin Büchsel, Ulrich Geisen, Clara Beckenkamp, Tobias Wengenmayer, Barbara Zieger, Dirk Westermann, Patrick M. Siegel

**Affiliations:** 1 Institute for Clinical Chemistry and Laboratory Medicine, Medical Center - University of Freiburg, Faculty of Medicine, University of Freiburg, Hugstetter Str. 55, 79106 Freiburg, Germany; 2 Interdisciplinary Medical Intensive Care (IMIT), Medical Center-University of Freiburg, Faculty of Medicine, University of Freiburg, Freiburg, Germany; 3 Department of Pediatrics and Adolescent Medicine, Division of Pediatric Hematology and Oncology, Faculty of Medicine, Medical Center–University of Freiburg, Freiburg, Germany; 4 Department of Cardiology and Angiology, University Heart Center Freiburg – Bad Krozingen, Faculty of Medicine, University of Freiburg, Freiburg, Germany

**Keywords:** Extracorporeal Membrane Oxygenation (ECMO), Von Willebrand factor (VWF), Von Willebrand Disease, Acquired von Willebrand syndrome, Platelet Function Analyzer (PFA), Bleeding, Intensive care

## Abstract

**Background:**

Extracorporeal membrane oxygenation (ECMO) is applied in patients with respiratory or cardiopulmonary failure, but bleeding is a frequent complication contributing to the high mortality rates in this patient collective. A major factor predisposing patients to bleeding events is an acquired von Willebrand syndrome (aVWS). So far, specific treatment options for this phenomenon are lacking. In hereditary von Willebrand disease (VWD), treatment with recombinant or plasma-derived von Willebrand factor (rVWF or pVWF) is common practice. Closure time measured by the Platelet Function Analyser-200 (PFA-200) is an established assay to detect defects in primary hemostasis and the method is useful to monitor the effect of hemostatic therapy. The aim of this study was to assess the effect of recombinant (rVWF) vs. plasma-derived von Willebrand factor (pVWF) on closure times measured by PFA in blood obtained from ECMO patients with aVWS.

**Methods:**

Blood was sampled from thirteen patients receiving extracorporeal membrane oxygenation and three patients with hereditary VWD. Diagnosis of aVWS was made by conventional coagulation parameters and by multimeric structure analysis. PFA analysis of blood spiked with rVWF or pVWF was performed.

**Results:**

Thirteen patients receiving ECMO were recruited. Ten patients survived and three patients suffered major bleeding complications. PFA closure times in ECMO patients with aVWS spiked with rVWF were significantly shorter at all concentrations than with pVWF (e.g., rVWF vs. pVWF: 1 U/ml: 150.4 ± 21.7 s vs. 263.8 ± 11.7 s; 4 U/ml: 97.8 ± 9.8 s vs. 195.8 ± 15.4 s, *p*<0.001). PFA closure times were also significantly shorter in three patients with hereditary VWD treated with rVWF compared to pVWF (e.g., 1 U/ml rVWF vs. pVWF: 73.7±1.33 s vs. 231.3±43.4 s, *p*<0.01)

**Conclusion:**

In summary, this study shows that rVWF compared to pVWF more effectively reduced PFA closures times in blood samples of ECMO patients with aVWS. Higher doses of VWF are needed to normalize PFA closure time in blood samples of patients with ECMO-induced aVWS compared to hereditary VWD. These data support the use of PFA-200 to monitor hemostatic effects in a future clinical trial involving ECMO patients with aVWS.

## Background

Extracorporeal membrane oxygenation (ECMO) is being increasingly used in patients with respiratory or cardiopulmonary failure [1]. Although many patients benefit from ECMO, mortality in patients receiving this treatment is high, ranging between 40-60 % depending on the cannulation strategy and underlying condition [2, 3]. Apart from the primary disease, bleeding is a leading cause of morbidity and mortality in ECMO patients. The so-called *ECMO-induced coagulopathy* is responsible for these bleeding events and has a complex pathogenesis [4, 5]. Exposure of blood components to high shear stress and large extracorporeal surfaces in the ECMO circuit affects multiple components of the coagulation system. For example, patients are predisposed to bleeding by the resulting ECMO-induced thrombocytopenia [6], platelet dysfunction [7], hyperfibrinolysis [8] and acquired von Willebrand syndrome (aVWS) [9, 10]. In particular, the aVWS is characterized by a loss of high molecular weight von Willebrand factor (VWF) multimers, which is most likely caused by shear stress in the ECMO circuit and possibly enhanced cleavage by a disintegrin and metalloproteinase with a thrombospondin type 1 motif, member 13 (ADAMTS13) [11]. Acquired VWS needs to be differentiated from the hereditary forms of von Willebrand disease (VWD) as it usually ceases after weaning from ECMO [9], whereas the hereditary forms are chronic diseases. Furthermore, there are established treatment options for the hereditary forms of VWD, for example, recombinant and plasma-derived VWF (rVWF and pVWF) [12, 13], but data for the treatment of aVWS in ECMO patients is insufficient.

Since VWF is essential for primary hemostasis, aVWS can be sensitively detected by the Platelet Function Analyzer PFA-200. Its advantages include simplicity, ease of execution and high sensitivity compared to measurement of bleeding time [14, 15].

The aim of this study was to assess the effect of recombinant vs. plasma-derived von Willebrand factor on closure times measured by PFA in blood obtained from ECMO patients with aVWS.

## Methods

### Patient recruitment and blood sampling

Patients receiving veno-arterial (VA) or veno-venous (VV) ECMO were recruited from the medical intensive care units of the University Hospital in Freiburg, Germany, from October until December 2021. One blood sample per patient was obtained within an interval 12-60 hours (h) after initiation of ECMO. As defined by the study protocol, the following exclusion criteria applied: hemoglobin (Hb) level <8 g/dl, platelet count < 100 G/l, hematological malignancies. All study participants were older than 18 years. Using an arterial line, blood was sampled slowly into tubes containing buffered 3.8% tri-sodium-citrate (Sarstedt, Germany)

Clinical and laboratory parameters from ECMO patients were gathered from the electronic patient data management system closest to the time point of blood sampling.

Major bleeding was defined as previously described [16]. Patients were counted as survivors if they were discharged from the intensive care unit.

As part of a limited case series, patients with hereditary VWD were also recruited by our local outpatient clinic. Blood was taken slowly into by antecubital vein puncture at one time point. Patients had not taken any medication in the past 14 days.

The buffered citrated whole blood was left untreated (“blank” or “untreated” sample), or supplemented with increasing concentrations (final concentration 0.5-4 U/ml) of recombinant (Vonicog alfa, Takeda Pharmaceutical, Japan) or plasma-derived VWF (Haemate ®, CSL Behring, Germany) (“spiked” whole blood). The assays were then carried out in these “untreated or spiked” samples.

### ECMO management

Nomenclature used in this paper is based on recommendations by the Extracorporeal Life Support Organization [17, 18]. Patients receiving ECMO were managed as previously described [7, 19, 20]. In brief, ECMO placement was indicated by an experienced ECMO physician. ECMO was placed by percutaneous cannulation. The configuration for VA ECMO was ‘V21-23_f_ - A15-17_f_‘ (femoral venous drainage to femoral arterial return; venous cannula diameters were 21-23 French (Fr), arterial cannula diameters were 15-17 Fr). The configuration for VV ECMO was ‘(dl27-31) Vj-V’ (dual-lumen jugular venous drainage to venous return; diameters ranging between 27-31 Fr).

If required, ECMO patients received red blood cell transfusions to maintain hemoglobin levels above 8 g/dl. Patients without signs of bleeding or thrombosis (including the ECMO circuit) were anticoagulated with unfractionated heparin aiming for an activated partial thromboplastin time (APTT) of 40-50 s. If patients showed signs of bleeding or thrombosis, individual targets for the APTT and platelet counts were decided.

The systems used for ECMO included the Maquet Cardiohelp System with an HLS Set Advanced (Maquet Cardiopulmonary GmbH, Rastatt, Germany), the Stöckert® centrifugal pump (LivaNova PLC, London, United Kingdom) and the Centrimag^TM^ system (Abbott Cardiovascular, Chicago, USA).

### Analysis of von Willebrand factor parameters

VWF activity was quantified in the central laboratory of the University Hospital in Freiburg using the INNOVANCE® VWF Ac (Siemens Healthineers, Germany) on a Sysmex CS5100 analyzer.

In addition, analysis of VWF antigen, VWF collagen binding capacity (VWF:CB) and VWF multimeric analysis were performed as previously described [21].

### Platelet Function Analyzer (PFA)

The PFA-200 (Siemens Healthineers, Germany) assay was carried out following the manufacturer’s instructions. Col/ADP cartridges (containing collagen and Adenosine diphosphate (ADP)) were used because they are insensitive to aspirin and nonsteroidal anti-inflammatory drugs (NSAID). If closure time was longer than exactly quantifiable, the longest time quantified by the instrument was used for further analysis. ‘Normalization’ after spiking of blood with rVWF or pVWF designates a closure time <121 s, which is the upper reference limit provided by the manufacturer.

Many PFA studies used the PFA-100. Therefore, it has to be mentioned that PFA-100 and PFA-200 use the same fundamental mechanics and deliver equivalent results [22].

### Ethics

This study was carried out in accordance with the declaration of Helsinki. All patients or their legal guardians provided informed consent. The study protocol for the recruitment of ECMO patients was approved by the Ethics Committee of the University Hospital in Freiburg, Germany.

### Statistics

Variables are presented as mean ± standard error of the mean (SEM) or as median (interquartile range) as indicated in the figure or table legend. One-way ANOVA followed by Tukey’s post-hoc analysis was performed to compare three or more unpaired variables. A *p*-value ≤0.05 was considered statistically significant. Analysis was performed using GraphPad Prism V.9.4.1 (GraphPad Software, San Diego, California, USA).

## Results

Thirteen patients receiving ECMO were recruited for this study (Table 1). Four patients were female, nine patients were male. Eleven patients were on VV ECMO, two patients were on VA ECMO. The underlying condition for the VV ECMO patients was ARDS, most commonly due to Coronavirus disease 2019 (COVID-19). One patient received VA ECMO for COVID-19 associated perimyocarditis and the other patient for COVID-19 associated pulmonary embolism.

**Table 1:** Clinical characteristics of ECMO patients

**Parameter**	**Value**
Patients	13
Age (y, IQR)	50.0 (34.5–53.5)
Female, n (%)	4 (31)
BMI (kg/m^2^, IQR)	31.0 (26.9-40.3)
Survivors, n (%)	10 (77)
SOFA Score (IQR)	10.0 (9.0-11.5)
Acute renal failure, n (%)	5 (38)
Dialysis, n (%)	1 (8)
Creatinine (mg/dl, IQR)	1.0 (0.6-3.4)
Urea (mg/dl, IQR)	91.0 (55.0-128.5)
Mechanical ventilation, n (%)	11 (85)
Respiratory rate (/min, IQR)	23.0 (14.5-26.5)
pH (IQR)	7.39 (7.35-7.44)
p_a_O_2_ (mmHg, IQR)	70.0 (64.3-79.9)
p_a_CO_2_ (mmHg, IQR)	45.6 (40.1-47.5)
Antibiotics, n (%)	8 (62)
Transfusions (Red Blood Cells), n (%)	2 (15)
COVID-19, n (%)	10 (77)
VV ECMO, n (%)	11 (85)
- ARDS, n (%)	11 (85)
◦ COVID-19 associated, n (% of ARDS)	9 (82)
VA ECMO, n (%)	2 (15)
- Pulmonary Embolism, n (%)	1 (8)
- COVID-19 associated Perimyocarditis, n (%)	1 (8)
Type of ECMO, n (%)	
- Stöckert Sorin	7 (54)
- Maquet	3 (23)
- Centrimag	3 (23)
Days on ECMO (d, IQR)	1.0 (1.0-2.0)
Blood flow (l/min, IQR)	4.0 (3.1-4.8)
FiO (%, IQR) in ECMO circuit	100 (90-100)
Noradrenalin, n (%)	9 (69)
Major bleeding, n (%)	3 (23)
- Intracerebral hemorrhage	2 (15)
Minor bleeding, n (%)	4 (31)
Heparin, n (%)	10 (77)
ASA, n (%)	1 (8)
Hemoglobin (g/dl, IQR)	9.9 (9.2-10.5)
Hematocrit (%, IQR)	29.4 (27.5-30.7)
Prothrombin time (s, IQR)	11.4 (11.0-14.1)
INR (IQR)	1.1 (1.0-1.2)
APTT (s, IQR)	48.0 (31.5-61.0)
Fibrinogen (mg/dl, IQR)	386.0 (234.0-607.5)
Platelets (x10^3^/µl, IQR)	225.0 (133.5-264.0)
CRP (mg/l, IQR)	62.9 (36.1-245.3)
PCT (ng/ml, IQR)	1.3 (0.2-3.6)
Leukocytes (x 10^3^/µl, IQR)	11.2 (7.6-15.0)
AST (U/l, IQR)	105.0 (44.5-190.5)
ALT (U/l, IQR)	50.0 (35.0-133.5)
Bilirubin (mg/dl, IQR)	1.1 (0.5-1.4)
Lactate (mmol/l, IQR)	1.3 (0.9-1.8)

Data are presented as median (interquartile range, Q1-Q3) or number of patients (%). Denominator of the percentage is the total number of subjects in the group. Clinical and laboratory parameters from individual ECMO patients were gathered from the electronic patient data management system closest to the time point of blood sampling. Therefore, they reflect the clinical situation at the time of blood sampling. Transfusions indicates the number of patients that received transfusions on the day before blood taking (only red blood cell transfusions were given). During the time on ECMO nearly all patients required transfusions. The bleeding complications presented occurred during the total time while patients were on ECMO and not at the time of blood sampling. Days on ECMO only reflect the first 60 hours of ECMO therapy during which blood was sampled. *ALT* Alanine aminotransferase, *ARDS* Acute respiratory distress syndrome, *ASA* Acetylsalicylic acid, *AST* Aspartate aminotransferase, *COVID-19* Coronavirus disease 2019, *BMI* Body mass index, *CRP* C-reactive protein, *F*_*i*_*O* Fraction of inspired oxygen, *Hb* Hemoglobin, *INR* International normalized ratio, *PCT* Procalcitonin, *APTT* Activated partial thromboplastin time, *VA ECMO* Veno-arterial extracorporeal membrane oxygenation, *VV ECMO* Veno-venous extracorporeal membrane oxygenation

Ten patients were discharged from intensive care and were counted as survivors. One patient died due to severe intracranial bleeding on ECMO. The other two patients died because of the severity of their underlying diseases. Major bleeding was observed in three patients and minor bleeding was documented in three patients. Two patients suffered from intracranial bleeding. Transfusions were required in two patients the day before blood sampling (red blood cells), but throughout their time on ECMO nearly all patients required transfusions. None of the patients required platelet transfusions the day before blood sampling.

Acquired VWS in ECMO patients was confirmed by pathological VWF activity to antigen ratios and markedly reduced or missing high molecular weight von Willebrand factor (HMW VWF) multimers (Table 2). All patients had prolonged PFA closure times and all but one patient had PFA closure times beyond the upper test limit. Platelet counts were > 100 G/l (as was required by the study protocol).

**Table 2:** Parameters of VWF function in ECMO patients

**Parameter**	**Value**	**Normal values**
VWF Antigen (IU/dl, IQR)	432 (327 - 728)	60 - 150
VWF Activity (IU/dl, IQR)	247.0 (182.0 - 382.5)	47.8 - 173.2
VWF Activity/Antigen (IQR)	0.51 (0.44 – 0.64)	0.73 - 1.16
VWF CBA (IU/dl, IQR)	120 (57 - 225)	60 - 150
VWF CBA/Antigen ratio (IQR)	0.24 (0.18 – 0.42)	0.8 – 1.5
HMW VWF multimers, n (%)	Reduced: 3 (23) Absent: 10 (77)	(normal)

Data are presented as median (interquartile range, Q1-Q3) or number of patients (%). Denominator of the percentage is the total number of subjects in the group (*n*=13). *CBA* Collagen binding activity, *HMW* High molecular weight

**Fig. 1:**
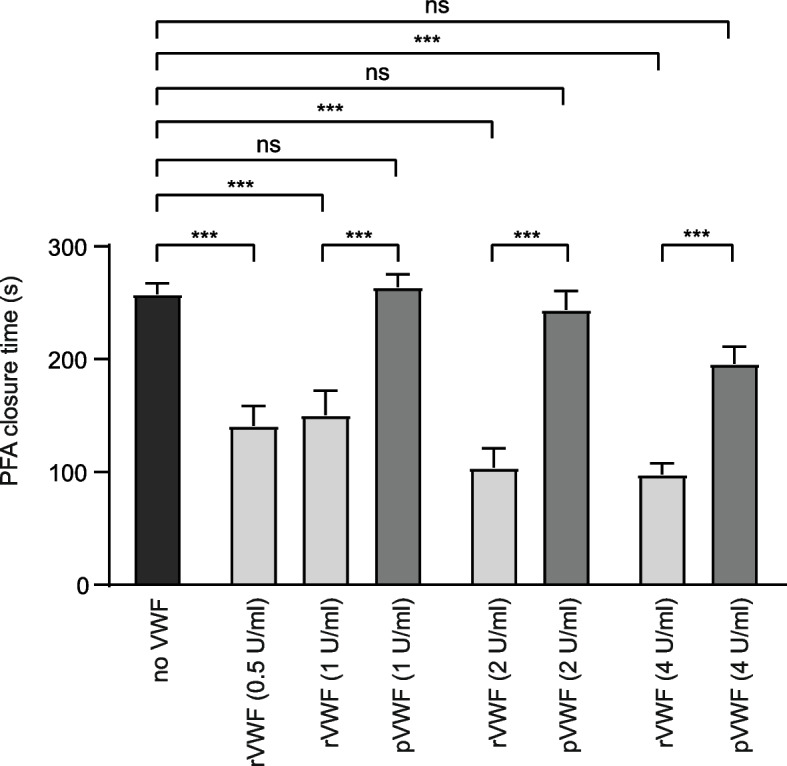
Effects of treatment with recombinant or plasma-derived VWF (rVWF vs. pVWF) on PFA COL/ADP closure time of samples from thirteen patients with ECMO-induced aVWS. The closure time was analyzed *in-vitro* on buffered citrate whole blood from ECMO patients spiked with rVWF or pVWF using the PFA-200 analyzer. If the time until hemostasis was longer than exactly quantifiable by the instrument, the highest value presented by the instrument was chosen for analysis. Data are presented as mean ± SEM. Data were analyzed by unpaired ANOVA. ns – not significant, ***p<0.001

We used the PFA-200 as an *in-vitro* model of primary hemostasis to estimate the effect of different VWF concentrates in the context of ECMO-induced aVWS. In citrated whole blood samples from ECMO patients which were “spiked” with either recombinant or plasmatic von Willebrand factor (VWF), recombinant and plasmatic VWF led to a dose dependent reduction of the PFA COL/ADP closure time (Fig. 1). Closure times were significantly lower if blood was spiked with rVWF vs. pVWF, which was the case for all concentrations (e.g., closure time (s), rVWF vs. pVWF: 1 U/ml: 150.4 ± 21.7 s vs. 263.8 ± 11.7 s; 4 U/ml: 97.8 ± 9.8 s vs. 195.8 ± 15.4 s, *p*<0.001). In summary, rVWF was significantly more effective in correcting a pathologic PFA closure time in blood samples from patients with ECMO-induced aVWS than pVWF.

**Fig. 2:**
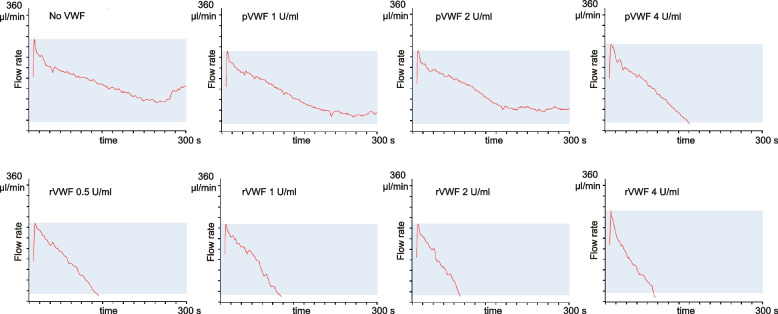
Evaluation of the PFA-200 COL/ADP closure curves. The flow rate in µl/min is monitored until either complete closure occurs, the measurement exceeds 300 s or the sample volume runs out (which can happen in samples with a low hematocrit). In samples from ECMO-induced aVWS a sudden incline in flow can occur, as seen in the present example for the whole blood sample without VWF after approx. 270 s

Figure 2 shows the PFA closure curves of samples from patients with aVWS. We also observed a reduction of the flow rate over time in untreated samples which can be interpreted as partial occlusion of the PFA capillary. There is a more pronounced reduction in the flow rate after addition of VWF, which does not always lead to PFA closure as it can be seen in Fig. 2 for the pVWF concentrations 1 U/ml and 2 IU/ml.

**Fig. 3:**
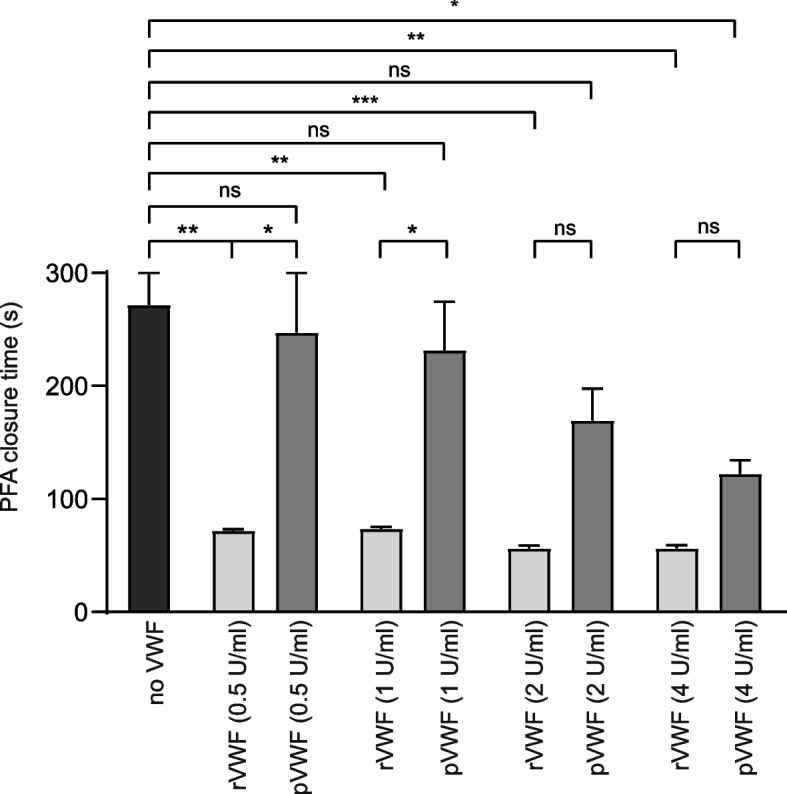
Effects of treatment with recombinant or plasma-derived VWF on PFA COL/ADP closure time of samples from patients with VWD. The closure time was analyzed *in-vitro* on buffered citrate whole blood from three VWD patients spiked with rVWF or pVWF using the PFA-200 analyzer. If the time until hemostasis was longer than exactly quantifiable by the instrument, the highest value presented by the instrument was chosen for analysis. Data are presented as mean ± SEM. Data were analyzed using unpaired ANOVA. *p<0.05, **p<0.01, ***p<0.001

After the unexpected finding that the dose of rVWF concentrate required to normalize the PFA closure time in aVWS was much higher than described for hereditary VWD [23], we assessed the effectiveness of rVWF vs. pVWF in a small group of patients with hereditary VWD (*n*=3). Two of these patients had VWD type 1 (one of them with Vicenza type VWD characterized by low VWF due to short VWF half-life) and one patient had type 2A VWD (formerly described as 2C Miami). VWD parameters (VWF activity and VWF antigen) were severely impaired (Table 3). We found that both, rVWF and pVWF, were able to normalize PFA closure times in spiked blood samples of patients with hereditary VWD, but rVWF was more effective in correcting the pathologic PFA closure time at lower dosages (e.g., 0.5 & 1 U/ ml) compared to pVWF (e.g., closure time (s) 1 U/ml rVWF vs. pVWF: 73.7±1.33 vs. 231.3±43.4, *p*<0.01, Fig. 3). The PFA closure time could be normalized in all samples from patients with hereditary VWD with the lowest rVWF dose tested (0.5 U/ml). However, for aVWS, higher dosages of rVWF were required to normalize PFA closure times: for example, in 10/13 (77 %) cases PFA closure times could be normalized with highest concentration of rVWF tested, but PFA closure time also improved from baseline in the remaining 3 patients that did not achieve normal PFA closure time. Therefore, we could confirm that the dose required to normalize the PFA closure is higher in ECMO-induced aVWS compared to VWD.

**Table 3:** Parameters of VWF function in hereditary VWD patients

**Patient**	**Diagnosis**	**VWF activity (IU/dl)**	**VWF antigen (IU/dl)**	**VWF Activity/Antigen ratio**
1	VWS type 1	42	39	0.93
2	VWS type 1 (Vicenza)	10	10	1
3	VWS type 2A (2C Miami)	166	14	0.08

Values are presented for each patient individually

Regarding the PFA closure curves, we frequently observed a sudden incline in the flow rate (Fig. 2, with no VWF) in aVWS patients which may correspond to instability in the primary thrombus. We did not observe this phenomenon when assessing samples from hereditary VWD patients.

## Discussion

Bleeding is a leading cause of morbidity and mortality in ECMO patients [5, 9]. Acquired von Willebrand syndrome (aVWS) contributes to the bleeding tendency observed in patients on ECMO support [4, 24]. In the current study, we recruited thirteen ECMO patients with ECMO-induced aVWS. Presence of aVWS was confirmed by VWF multimeric structure analysis. Major bleeding was observed in three patients, two of them suffered from intracranial hemorrhage. Although bleeding is frequent in ECMO it has yet to be determined how to manage aVWS-related bleeding in ECMO patients [5]. After discontinuation of ECMO, HMW VWF multimers are restored within 6 hours [9]. However, if ECMO cannot be discontinued, Desmopressin (DDAVP) is an option in some patients, however, Desmopressin resistance during ECMO due to exhausted endothelial storage sites can occur. This phenomenon has recently been described in COVID-19 patients on ECMO support [25]. Especially in this context, therapy with VWF concentrates has to be considered. In this study, we compared the potential of different VWF concentrates in an in-vitro model.

PFA closure time is an established assay to detect defects in primary hemostasis [26, 27] and the method is useful to monitor the effect of hemostatic therapy for example with DDAVP [28, 29, 30]. After whole blood samples from patients with ECMO-induced aVWS had been spiked with increasing concentrations of rVWF or pVWF, we observed a dose dependent reduction of the PFA closure time. As there are differences in the HMW VWF multimer content of different plasma-derived VWF concentrates, the concentrate which had the highest HMW multimer content in a comparison of 12 concentrates was used [31]. Nevertheless, rVWF was significantly better than pVWF in normalizing the PFA closure time of whole blood samples from patients with aVWS. It is known that rVWF and pVWF differ in their multimer composition [32]. The rVWF contains ultra-large (UL) VWF multimers because it has not been in contact with ADAMTS13. Our findings are in line with a recently published comparison of rVWF and pVWF in ECMO patients that showed a better effect of rVWF on ristocetin-induced platelet aggregation (RIPA) [33].

Unlike most pVWF concentrates, rVWF does not contain factor VIII which can be very high in COVID-19 patients [34, 35]. Critically ill patients with COVID-19 require ECMO relatively often if an acute respiratory distress syndrome develops [36]. It has recently been shown that pVWF concentrates were able to enhance VWF activity in ECMO-induced aVWS patients [37]. The mean VWF ristocetin cofactor activity to antigen ratio increased after treatment but was not normalized. Control of bleeding within 24 hours was achieved in 4/10 patients. *In-vivo* data regarding the use of rVWF in ECMO aVWS are still missing. Therefore, more in-vivo studies regarding treatment of ECMO-induced aVWS are elementary.

It is difficult to monitor the treatment of the so-called ‘type 2 aVWS’ (as most often observed in ECMO-induced aVWS) since these patients already have very high VWF levels [34, 35]. Unlike for patients with hereditary VWD there is no well-defined target range for substitution in bleeding situations. Our data indicate that the PFA closure time may be useful in monitoring the effect of VWF concentrates in situations of already elevated VWF levels.

One PFA study showed a dose-dependent reduction of PFA closure time in patients with hereditary VWD by spiking buffered citrated whole blood with recombinant von Willebrand factor (rVWF) [23]. In their study with 12 patients suffering from hereditary VWD, Pekrul et al. observed a normalization of PFA closure time in all blood samples after spiking in 1 U/ml rVWF which was not sufficient in many samples from patients with aVWS in our study. To investigate if in aVWS higher doses of VWF may be needed to normalize PFA closure times we investigated the effect of rVWF and pVWF on PFA closure time in spiked blood samples from patients with VWD. We observed a dose dependent reduction in PFA closure time for pVWF and rVWF. In line with the literature, a concentration of 0.5 U/ml rVWF was sufficient to normalize PFA closure time in the context of hereditary VWD [23]. When we looked at the PFA closure curves (Fig. 2) a sudden incline in the flow rate could be observed in some samples from aVWS patients but not from VWD patients. This could be interpreted as a sign for instability in the primary thrombus occluding the PFA capillary. There were some important differences between samples from aVWS and VWD. The samples from aVWS patients had higher baseline VWF antigen levels, lower platelet counts (although all had a platelet count > 100.000/µl) and a lower hematocrit. Both the patients’ VWF and the added VWF can competitively bind to collagen inside the PFA capillary. A competing effect of abundant VWF from the patient might theoretically reduce the effect of spiked VWF. However, we found no correlation between VWF antigen level and PFA closure time. Low platelet count and low hematocrit might also lead to prolonged PFA closure time. However, no significant correlation between PFA closure time and platelet count or hematocrit was observed. The severity of platelet dysfunction [7, 9] might also affect the PFA closure time but was not assessed in the current study. In conclusion, our model predicts a worse response to VWF substitution in ECMO-induced aVWS than in VWD as it has been described for other forms of aVWS [38].

Our study has some limitations. First, it has yet to be shown that the results of our *in-vitro* study hold true for the *in-vivo* situation. Most of the patients were on ECMO due to COVID-19. Therefore, the results might not be generalizable to ECMO patients in general. Although in the small subset of non-COVID patients on ECMO the results were consistent. There are factors other than VWF that affect PFA closure time. We consider platelet count, hematocrit and platelet dysfunction to be the most relevant in the current study. But also drugs [39], ABO blood group and time of blood collection [40] are known confounders of PFA closure time. To avoid interference by aspirin and NSAID, Col/ADP cartridges were used. One blood sample per patient was obtained within a 12-60 hours interval after ECMO initiation and not at exactly the same time point which due to the variation in time on ECMO may have had an effect on the severity of the underlying aVWS and therefore could have influenced PFA results.

In summary, we showed that different VWF concentrates reduced the PFA closure time in a dose dependent manner. Interestingly rVWF was more effective than pVWF in normalizing PFA closure time. Higher doses of VWF were needed to normalize PFA closure time in ECMO aVWS compared to hereditary VWD. These data support PFA-200 to monitor hemostatic effects in a future clinical trial involving ECMO patients with aVWS. *In-vivo* studies will give more insights regarding the effectiveness of different therapies.

## Data Availability

The datasets used and/or analyzed during the current study are available from the corresponding author upon reasonable request.

## Abbreviations

ADAMTS13: A disintegrin and metalloproteinase with a thrombospondin type 1 motif, member 13
ADP: Adenosine diphosphate
APTT: Activated partial thromboplastin time
aVWS: Acquired von Willebrand syndrome
COL: Collagen
CBA: Collagen binding activity
COVID-19: Coronavirus disease 2019
DDAVP: Desmopressin
ECMO: Extracorporeal Membrane Oxygenation
Fr: French
Hb: Hemoglobin
HMW: High molecular weight
Hb: Hemoglobin
IQR: Interquartile range
NSAID: Nonsteroidal anti-inflammatory drugs
PFA: Platelet function analyzer
pVWF: Plasma-derived von Willebrand factor
RIPA: Ristocetin-induced platelet aggregation
rVWF: Recombinant von Willebrand factor
SEM: Standard error of the mean
VA: Veno-arterial
VV: Veno-venous
VWD: von Willebrand disease
VWF: von Willebrand factor
